# Construction of digital competency model of moral education teachers in colleges and universities based on smart learning environment: an empirical study based on mixed method

**DOI:** 10.3389/fpsyg.2026.1834084

**Published:** 2026-07-03

**Authors:** Zhongyuan Liao

**Affiliations:** School of Education, Fuzhou University of International Studies and Trade, Fuzhou, China

**Keywords:** digital competency, mixed methods research, moral education teachers, smart learning environment, structural equation modeling, teacher professional development

## Abstract

**Introduction:**

This study developed a new digital competency model specifically for university moral education teachers working in smart learning environments. While most existing research focuses on general digital skills, this study addresses the unique challenge of how moral education teachers can effectively integrate technology while maintaining focus on values-based educational goals.

**Methods:**

Using a mixed-method approach, the researchers first conducted behavioral event interviews with 50 teachers and identified 37 competency indicators through grounded theory analysis. These indicators formed a five-dimension theoretical model: value-oriented digital literacy, technology integration capability, digital pedagogical competence, educational innovative thinking, and digital citizenship leadership. The model was then validated through 292 questionnaires. After statistical analysis, a formal 34-item scale was developed.

**Results:**

Confirmatory factor analysis showed good model fit indices, confirming the structural validity of the competency framework. A key finding was that value-oriented digital literacy carried the highest weight among all dimensions, emphasizing that ethical considerations and value judgments are fundamental when moral education teachers use technology.

**Discussion:**

This highlights the distinctive nature of moral education compared to other subjects, where technical skills might be prioritized. The study makes important theoretical contributions by integrating value dimensions into digital competency frameworks, which are often missing from international generic models like DigCompEdu. Practically, it provides universities with a structured tool for designing training programs, developing curricula, and creating evaluation systems specifically for moral education teachers. While the model is grounded in the Chinese moral education context, its theoretical framework may inform similar research in other settings pending cross cultural validation. The findings also offer valuable references for promoting digital transformation in moral education across primary, secondary, and tertiary levels.

## Introduction

Digital transformation is profoundly reshaping the landscape of higher education. Smart learning environments with artificial intelligence, big data, learning analysis and other technologies as the core not only provide new tools for optimizing knowledge transfer and skill training, but also put forward new requirements for cultivating learners’ higher-order thinking, critical judgment and responsible citizenship, and also bring new challenges (Liu et al., 2025). In this study, a “smart learning environment” is defined as a technology-enhanced educational setting with three interconnected features. First, it involves intelligent technological intermediation, meaning that technologies such as artificial intelligence, learning analytics, and cloud computing are deeply embedded throughout the entire instructional process from content delivery to assessment. Second, it features data-driven personalization, whereby the environment continuously collects and analyzes learner data to adapt instruction to individual students’ progress and needs. Third, it provides context-aware support for higher-order thinking, as the environment is designed to foster critical thinking, ethical reasoning, and meaning negotiation rather than merely delivering information or basic skills. These three features distinguish a smart learning environment from general digital learning environments, such as those relying solely on online resource repositories or basic ICT tools, which often lack real-time analytics, adaptive feedback, or a pedagogical focus on higher-order cognitive development. In the humanities and social sciences, the teaching of philosophy, history, civic education and other subjects has a distinct value orientation and educational attributes. The teaching content of this type of course must follow the rules of students’ cognition and growth, and is committed to guiding students to conduct value analysis, ethical reflection and identity. The teaching process is essentially a complex process of meaning negotiation. In a smart learning environment, teachers are the active subjects in realizing technology-empowered educational changes. For the teaching of moral education subjects, teachers not only need to master general digital teaching skills, but also need to develop a special ability that can effectively guide students to engage in critical thinking, ethical discussion and meaning construction in a digital environment mediated by technology and complicated information. As UNESCO points out, digital technology contains huge potential for educational transformation, but the key is to find an effective path to transform it into real educational power (UNESCO, 2021). Among them, developing teachers’ digital competency is an important way to transform technological potential and cope with the above-mentioned teaching complexity (Wu et al., 2025). It can be said that the digital competency of moral education teachers in colleges and universities has become a key capability that empowers the integrated construction of moral education courses in universities, middle schools and primary schools. The particularity of moral education teaching in colleges and universities under a smart learning environment is that technology is not only a teaching tool, but also an intermediary that deeply reconstructs teacher-student interaction, knowledge presentation, and meaning negotiation. In this environment, the value issues, ethical dilemmas and cultural perspectives involved in teaching constitute important factors affecting the selection and application of technology. Therefore, this study regards this type of value situation as a key variable that affects technology integration and teaching practice, rather than an isolated digital competency dimension, and explores how a teacher digital competency model should be constructed to effectively respond to the needs of high-level teaching objectives while maintaining a dialogue space with general educational technology theory.

Existing research has laid a solid foundation for understanding teachers’ digital competence. Internationally adopted digital competence frameworks, such as the European Union’s DigCompEdu, provide a common reference for assessing educators’ technological capabilities (Li et al., 2024; Licen and Prosen, 2024). Studies indicate that teachers’ digital competence demonstrates significant correlations with their attitudes toward technology integration (Galindo-Domínguez et al., 2024), autonomous support in instruction (Chen et al., 2024), and even the cultivation of students’ digital citizenship literacy (Xu et al., 2025). However, most current research still focuses on technology integration within STEM disciplines or general teaching contexts (Althubyani, 2024), while empirical investigations into effective technological applications within humanities and social sciences education under smart learning environments remain insufficient (McGarr, 2024). Smart learning environments aim to foster higher-order thinking and innovation abilities through intelligent resource delivery and process data analysis (Horváth et al., 2025), which presents new demands on teachers’ competency structures. Nevertheless, related empirical studies—particularly in moral education domains—remain limited. Although existing research has identified factors such as school support (Chiu et al., 2024), professional identity (Li et al., 2025), and psychological capital (Yan et al., 2025) as influential to teacher development, how these factors interact with moral education curriculum objectives within smart learning environments to collectively shape teachers’ professional competence remains an unexplored “black box” requiring further investigation.

In order to fill the above research gap, this study aims to construct and verify a digital competency model for college moral education teachers suitable for smart learning environments. The specific research questions are as follows: (1) What are the core components of the competency of college moral education teachers in a smart learning environment? (2) What is the structural relationship among these elements? (3) Can this model effectively predict or explain the digital competency level of moral education teachers? This study builds a scientific, systematic and situational competency model to provide reference for establishing digital development goals, research and training systems, and formulation of evaluation standards for moral education teachers in colleges and universities, thereby enriching the theory of teacher professional development in the context of smart education.

## Literature review

The theoretical evolution of teachers’ digital competence clearly demonstrates a trajectory shifting from general competency constructs toward contextualized practices. In 1973, McClelland first proposed the concept of “competence,” advocating for competency characteristic assessments to replace traditional intelligence tests such as academic performance, intellectual capacity, and aptitude measurements (McClelland, 1973). Spencer’s Iceberg Model further differentiated this into explicit components of “knowledge and skills” and implicit personal elements including “motivation, traits, and self-concept” (Spencer and Spencer, 1993). Within educational research, these classical theories established foundational frameworks for understanding the composite structure of knowledge, skills, and values within teacher professional quality (Olson and Wyett, 2000; Tigelaar et al., 2004). This conceptual evolution signifies a shift in research focus from universal human resource theories to in-depth analyses of the complex internal structures of teacher professional competencies, while simultaneously laying the theoretical groundwork for transforming teacher capability structures in the digital era.

In the 21st century, the connotation of digital competence has transitioned from operational skills to higher-order professional literacy. It is no longer merely a component of digital citizenship but has evolved into a core element of teachers’ professional competence (Castelfranchi, 2007), representing an integrated capability encompassing critical thinking, creative expression, and responsible action within complex digital environments (Ala-Mutka, 2011). Meanwhile, international mainstream framework development demonstrates diversification trends. For instance, the European Union’s “Digital Competence Framework for Educators” (DigCompEdu) outlines structured competency spectra and development pathways required for teachers’ professional activities in digital contexts (Redecker and Punie, 2017). The TPACK framework theoretically emphasizes that effective technology integration transcends simple tool superposition, instead constituting a dynamic, contextualized reconstruction process among technological knowledge (TK), pedagogical knowledge (PK), and content knowledge (CK) (Mishra and Koehler, 2006). In other words, teacher capacity development must achieve deep alignment with specific disciplinary logics and instructional objectives. This trend is corroborated by educational technology reforms in various countries: Australia embeds information and communication technology (ICT) competencies within its national teacher career development standards (AITSL, 2011); the UK’s Digital Teaching Professional Framework closely aligns with practical teaching field contexts (ETF, 2019); Ireland’s school digital strategy advances through four thematic pillars (DES, 2015). Falloon’s research further critiques traditional narrow conceptions of digital literacy, advocating for a more comprehensive and holistic approach to teacher digital competence (Falloon, 2020). These converging frameworks indicate that digital competence is no longer an isolated “add-on” within conventional teacher competency models, but rather constitutes a complex construct integrating “critical digital literacy” (Hinrichsen and Coombs, 2013) and “reflective practice” (Fisher et al., 2012) as advanced professional capabilities. Globally, teacher digital competence development faces challenges transitioning from “generic frameworks” toward “contextualized adaptation.” The effectiveness of digital competence is inherently highly context-dependent, with significant variations across disciplines regarding interpretations of digital tools’ nature, pedagogical approaches, and required teacher capabilities (Starkey et al., 2023). Its application extends beyond generic educational settings to being redefined and enacted within specific disciplinary cultures and emerging technological ecosystems (Markauskaite et al., 2023). As a technology-mediated educational environment, smart learning environments are characterized by techno-mediation permeating throughout the entire instructional process (Huang, 2008). At the cognitive level, technology acts as an intermediary to concretely understand abstract concepts through visualization tools, virtual simulations and other means; at the interactive level, learning management systems and collaboration platforms use intermediary properties to promote social interaction between teachers and students; at the evaluation level, learning analysis tools play an intermediary role to assist in the generation of teaching feedback and the adjustment of personalized learning paths. In this environment, technology is not only a channel for transmitting information, but also a lubricant that reconstructs the implementation of teaching methods, the organization of learning activities, and the way teachers and students interact with content (Newby and Cheng, 2020). This intermediary effect changes the relationship between teachers, content, and students in traditional teaching, requiring teachers to develop new “intermediate teaching capabilities” to effectively control the teaching possibilities and complexity brought by technology (Starcic et al., 2016). This aligns with the higher-order stages of “Modification” and “Redefinition” as defined in the SAMR model. In fact, moral education courses in colleges and universities constitute a teaching situation where value orientation gives priority. The teaching goal of this type of course is no longer just the acquisition of knowledge and skills, but also directly points to higher-level educational goals such as value shaping, critical thinking cultivation, and citizenship identity. Instead, teachers are required to use digital technology as a dialogue medium to promote meaning negotiation, ethical discussion, and value construction. However, existing research, whether exploring teacher competency (Ng et al., 2023) or digital leadership (Hoang, 2025) in the context of artificial intelligence, has yet to theorize digital teaching practices in this high-value educational context. This expertise requires teachers to always pursue the ultimate value of education in the application of technology, and to develop a matching digital practice ability system that integrates ethical considerations, cultural sensitivity and teaching wisdom.

As demonstrated above, although generic models and international frameworks for teachers’ digital competence have become increasingly mature, significant limitations emerge when these frameworks are examined within the disciplinary context of moral education in higher education institutions and the technological field of smart learning environments. Current research has failed to address a critical question: What unique, context-specific digital competencies should moral education teachers in higher education possess within smart teaching environments? In response to this gap, based on classical competency theory and drawing structural insights from international frameworks such as DigCompEdu, TPACK, and SAMR, this study attempts to construct a contextualized, discipline-specific digital competence model for moral education teachers that embeds ethical values. This model is developed through systematic integration of the techno-mediating characteristics of smart learning environments with the inherent pedagogical principles of moral education curriculum. Such an endeavor not only represents a valuable theoretical exploration into deepening the understanding of digital competence among moral education teachers, but also provides potential pathways for capacity development to achieve advanced humanistic educational objectives within complex technological environments.

## Methodology

### Method selection

Currently, common approaches to constructing teacher competency models include literature analysis, expert evaluation, questionnaire surveys, behavioral event interviews, and grounded theory. Among these methods, the integration of grounded theory and behavioral event interviews is widely recognized as an effective tool for uncovering teachers’ digital competencies, particularly in identifying key differences between exceptional and average performance. The behavioral event interview method, based on the interviewees’ job positions, systematically guides participants to recall and describe specific critical incidents—both their most successful and unsuccessful experiences at work. This process deeply explores the Situation, Task, Action, Result (STAR) framework along with personal reflections to extract latent competency characteristics. However, data collection alone is insufficient for developing a convincing theoretical framework. Grounded theory, which operates without pre-determined conclusions or hypotheses, employs three-level coding to iteratively compare, conceptualize, and categorize qualitative research materials, ultimately generating a theoretical framework that reflects the essence of social phenomena. This approach is particularly suitable for in-depth analysis of unstructured data like interview transcripts, effectively addressing “what” and “why” research questions. Therefore, this study first employs behavioral event interviews to collect vivid, concrete narrative data from moral education teachers regarding their experiences in smart teaching scenarios. Subsequently, following grounded theory’s rigorous analytical procedures, these data are coded and refined to reveal new requirements imposed by smart learning environments on moral education teachers’ digital competencies, thereby establishing a preliminary theoretical model framework. During the quantitative validation phase, to test and refine this initial model, the study references international standards including DigCompEdu and the “Teacher Digital Literacy” industry guidelines. A Likert 5-point scale is developed to create an initial measurement instrument. SPSS 26.0 is employed for item analysis, exploratory factor analysis (EFA), and reliability analysis, while AMOS 24.0 conducts confirmatory factor analysis (CFA) and model fit testing. Principal component analysis further calculates dimension weights and indicator coefficients. The final objective is to construct a theoretically robust digital competency model rooted in Chinese higher education moral education practices, offering strong explanatory power for understanding teacher capabilities in digital contexts.

### Research subjects

This study adopts a mixed sampling method that combines theoretical sampling and stratified sampling to ensure that the sample can cover various changes in the research phenomenon to the greatest extent and achieve theoretical saturation. In terms of sampling design, based on the stratified framework of institutions of higher education in China, moral education teachers from 12 universities at different levels in Fujian Province, China, were selected as the research subjects. While the sample was geographically limited to Fujian Province, the study employed theoretical sampling and stratified coverage of different institutional levels to achieve theoretical saturation, and included both target and control groups to isolate context specific competencies. This ensures that the sample structure reflects the diversity of Chinese universities and focuses on value-sensitive teaching situations. Based on the comparative paradigm of classic competency research, the interviewed teachers were divided into a target group and a control group to highlight the unique abilities required to adapt to the smart environment through comparison. The teachers in the control group refer to teachers in the traditional face-to-face classroom teaching environment who are recognized by the college or students as having excellent teaching effects, serious teaching attitude, and being loved by students. However, their application of smart teaching technology is still in the preliminary understanding or occasional trial stage, and has not been systematically integrated. This group serves as a frame of reference and aims to peel off general teaching competencies through comparison with the target group. The target group of teachers refers to teachers who highly identify with smart teaching in terms of teaching concepts; in the past 2 years of teaching practice, they have used at least one mainstream smart teaching platform or tool regularly and innovatively, such as Rain Classroom, Xuexitong, Moodle and others; Teachers’ smart teaching practices have won teaching awards at the school level or above, or have been widely praised in student evaluations and peer reviews. At the same time, the study controlled demographic variables such as gender, age, teaching experience, professional title, and academic qualifications of the two groups of teachers to reduce potential bias. In terms of sample size, 50 teachers who met the above criteria were recruited to participate in in-depth interviews in order to achieve theoretical saturation. Among them, 25 were in the target group and 25 were in the control group. All participants gave informed consent to participate in the study and were informed that they could withdraw at any time. The distribution of basic demographic characteristics of the participants is shown in Table 1.

**Table 1:** Basic composition information of interview subjects.

Category	Sub-item	Quantity	Proportion
Gender	Male	21	42%
	Female	29	58%
Age	Under 45	30	60%
	45 and above	20	40%
Teaching experience	0–5 years	8	16%
	6–20 years	20	40%
	20 years	22	44%
Professional title	Lecturer	9	18%
	Associate Professor	25	50%
	Professor	16	32%
Educational degree	Doctoral degree	30	60%
	Master degree	13	26%
	Undergraduate degree	7	24%

### Data collection and organization

This study, based on its research objectives and referencing McClelland’s classical competency interview question design principles (Olson and Wyett, 2000), integrated the requirements of moral education discipline teaching in terms of critical thinking, theoretical depth, and approachability. Ultimately, the “Critical Incident Interview Protocol for Digital Competency of University Moral Education Teachers in Smart Learning Environments” was established. The interview protocol comprises three modules: (1) Conceptual definitions to help teachers understand the research context, including smart learning environments and teacher digital competency; (2) Basic information collection covering name, gender, years of teaching experience, academic rank, workplace, taught courses, and research fields; (3) Core questions focusing on critical incidents related to teachers’ successes and failures in smart teaching practices. A semi-structured interview format was adopted, conducted individually through face-to-face interviews primarily supplemented by online video interviews, ensuring adequate interaction depth and duration of 40–100 min per session. Prior to formal data collection, pilot interviews were conducted with four teachers to test the protocol’s applicability and clarity, followed by adjustments based on feedback. The design logic and theoretical foundations of the main interview questions are presented in Table 2. All interviews were audio-recorded after obtaining informed consent. Additionally, selected high-quality online course videos, lecture recordings, and other instructional materials provided by participants served as supplementary details and triangulation validation sources. Subsequently, using the qualitative analysis tool NVivo 12, interview recordings were transcribed into textual data. During transcription, personal biases were strictly avoided to maintain factual accuracy, with meticulous documentation of both verbal content and non-verbal cues. This process yielded 50 transcripts totaling approximately 250,000 Chinese characters, providing a substantial raw data foundation for subsequent grounded theory analysis.

**Table 2:** Logical correlation of digital competency interview outline for ideological and political course teachers in colleges and universities.

Interview topic	Interview questions	Purpose of interview	Theoretical basis
Digital environment coping	Please recall a specific situation in which you had to quickly master and use a new digital tool to complete important ideological and political teaching tasks. What happened? What specific steps did you take? What was the result?	Evaluate teachers’ learning adaptability, problem-solving strategies, and action capabilities in the face of technological changes.	Based on critical digital literacy theory and value education theory, it focuses on teachers’ ability to guide students to conduct critical evaluation, ethical analysis and meaning negotiation in situations with multiple information and viewpoints.
Values leadership challenge	Please describe a situation in a digital environment where students had confusion or even extreme remarks regarding values or political views. How did you think and respond at the time? What impact did the results have on students’ minds?	Discover teachers’ core abilities and behavior patterns in value guidance and ideological correction in complex digital information environments.	Corresponding to the “critical evaluation” and “meaning negotiation” abilities in critical digital literacy theory, it emphasizes teachers’ professional judgment and educational intervention in situations of multiple value conflicts.
Teaching innovation breakthrough	Please tell us about one of your most successful examples of deeply integrating digital technology into the teaching content design of ideological and political courses. How did you come up with this idea? How to coordinate technology, content, and student interaction during implementation? Which capabilities played a key role?	Identify teachers’ innovative thinking, design capabilities, and key actions that deeply integrate digital technology with ideological and political education.	Based on the integration of “technological pedagogical content knowledge” within the TPACK framework, this study focuses on how teachers creatively integrate technology, pedagogy, and subject matter content in specific instructional contexts.
Crisis handling incident	Please share an unexpected technical failure or public opinion incident that occurred during the digital teaching process. How did you feel? What specific measures have been taken to control the situation and maintain teaching? What lessons were learned from this?	Explore teachers’ practical abilities in emergency response, emotion management, and crisis resolution in the digital environment.	Derived from the concept of “crisis leadership” in educational leadership research, it focuses on teachers’ emotional adjustment and emergency decision-making abilities in an environment of technological uncertainty.
Lasting impact incident	Please recall an incident that had a profound and positive impact on the intellectual growth of students through digital teaching methods. What special things have you done to make a difference in your students? What enduring beliefs or traits does this reflect on you?	Discover the deep motivations behind teachers’ behaviors, educational beliefs, and success factors that have a long-term impact on students.	This study investigates the theoretical connection between teachers’ professional identity and the long-term mechanisms of values education, while exploring key factors influencing sustained value internalization in digital teaching contexts.

To ensure the reliability and validity of the coding process, grounded theory analysis was independently conducted by two researchers trained in rigorous qualitative research methods. Prior to commencing coding, they received training on theoretical frameworks and coding protocols, and standardized label usage criteria and conceptual understanding through independent trial coding and iterative discussions. Subsequently, the two coders independently performed sentence-by-sentence analysis on 50 interview transcripts to extract initial concepts. Weekly consultation meetings combined with discrepancy resolution mechanisms were implemented to maintain concept labels’ proximity to raw data. Initial concepts were grouped into categories, while visual tools were employed to identify logical relationships between categories. Through continuous comparative analysis, core categories were identified to construct a theoretical narrative until theoretical saturation was achieved. As shown in Table 3, all texts demonstrated categorization consistency coefficients (CA) ranging from 0.679 to 0.783, with coding reliability coefficients (*R*) varying between 0.809 and 0.878. According to conventional reliability standards (*R* > 0.80 indicates excellent reliability), all *R* values exceeded 0.80, confirming that the coding results achieved excellent intercoder consistency and the coding process maintained stability and reliability (Hair et al., 1995). This established a solid and credible analytical foundation for subsequent theory construction.

**Table 3:** Coding coefficient table(R).

Interview text number	CA	R
1	0.682	0.811
2	0.715	0.834
3	0.701	0.825
4	0.691	0.818
5	0.724	0.840
6	0.731	0.845
7	0.77	0.870
8	0.721	0.838
9	0.683	0.812
10	0.756	0.861
11	0.726	0.841
12	0.708	0.829
13	0.784	0.879
14	0.713	0.832
15	0.717	0.835
16	0.776	0.874
17	0.685	0.814
18	0.719	0.837
19	0.782	0.878
20	0.785	0.880
21	0.738	0.850
22	0.702	0.825
23	0.751	0.858
24	0.763	0.866
25	0.722	0.839
26	0.74	0.851
27	0.752	0.858
28	0.728	0.843
29	0.781	0.877
30	0.762	0.865
31	0.714	0.833
32	0.749	0.856
33	0.733	0.846
34	0.779	0.876
35	0.705	0.827
36	0.788	0.881
37	0.721	0.838
38	0.759	0.863
39	0.742	0.852
40	0.774	0.873
41	0.698	0.822
42	0.736	0.848
43	0.753	0.859
44	0.725	0.841
45	0.711	0.831
46	0.769	0.869
47	0.732	0.845
48	0.744	0.854
49	0.716	0.834
50	0.791	0.883

This study ensures the rigor of the research process through researcher reflection and ethical practice. The research team consists of members with backgrounds in educational technology, moral education and psychology. In order to avoid potential researcher bias, this study adopts a number of control strategies: triangulation runs through the entire process of data collection, analysis and theory construction; writing reflective logs and conducting team discussions to continuously examine one’s own research stance and potential assumptions; preliminary research conclusions are returned to participants for member testing to ensure that theoretical explanations are close to teaching practice. In addition, Additionally, external experts were invited to conduct an independent academic review of the coding framework and research conclusions. At the ethical level, the principles of informed consent, confidentiality, data anonymization and results feedback were strictly followed. These measures jointly enhance the credibility and explanatory validity of this study, allowing the theoretical construction to be rooted in real and complex moral education teaching situations.

## Results

### Open coding

By reading, analyzing and conceptualizing sentence by sentence 50 in-depth interviews with college moral education teachers, a total of 412 valid original expressions were extracted. After comparison, labeling and induction, 112 themes were initially formed and further condensed into 49 initial conceptual categories. Secondly, by comparing the frequency of coding data of the two groups of teachers, and based on the professional standards and research consensus of moral education teachers, 14 categories with frequencies of 29 and above, which highly summarize the general professional qualities of moral education teachers, were defined as common competency indicators. Finally, the focus was on 35 discriminative digital competency index elements and their frequency distribution for college moral education teachers in a smart learning environment (as shown in Table 4).

**Table 4:** Frequency analysis table of elements of teachers’ digital competency index.

Type serial	Number	Indicator elements	Frequency
Common competency indicator elements	1	Discipline value recognition	59
	2	Discipline theoretical literacy	52
	3	Teaching planning ability	43
	4	Clear teaching logic	35
	5	Group collaboration ability	43
	6	Smooth expression and communication	40
	7	Professional responsibility	42
	8	Understand students’ thoughts	41
	9	Classroom norm awareness	38
	10	Teaching reflection and improvement	36
	11	Teaching appeal	39
	12	Professional knowledge base	29
	13	Psychological quality	30
	14	Awareness of academic norms	31
Discriminating competency indicator elements	1	Digital environment value sensitivity	22
	2	digital teaching efficacy	20
	3	Ability to critically evaluate network information	15
	4	Digital lifelong Learning ability	18
	5	Digital resource development ability	16
	6	Hidden discourse analysis ability	20
	7	Technical platform application ability	23
	8	Educational digital content creation	22
	9	Teaching data analysis ability	22
	10	Theoretical visualization ability	18
	11	Technology integration teaching design capabilities	17
	12	The public transformation power of academic discourse	19
	13	Classroom Organization and Guidance Ability	21
	14	Teaching Assessment Reflection Ability	16
	15	Youth Cultural Symbol Borrowing Ability	16
	16	Virtual Simulation Resource Development Ability	18
	17	Digital technology sensitivity	20
	18	Digital Humanities Narrative Expression	21
	19	Digital cultural transformation ability	14
	20	Innovation of technology-integrated teaching model	12
	21	Cloud Collaboration Management Ability	18
	22	Human-computer collaborative teaching design capabilities	10
	23	Critical thinking ability on problems	7
	24	Online classroom scheduling ability	17
	25	The influence of online academic discourse	6
	26	Network education community building power	20
	27	Digital pattern creativity	9
	28	Cross-cultural digital comparison	17
	29	Digital teacher ethics demonstration ability	5
	30	Digital ethical judgment	15
	31	Digital Ethics and Security Practice Ability	21
	32	Historical Context Creation Ability	18
	33	Classroom Interaction Value Assessment Ability	19
	34	Learning Behavior Thought Analysis Ability	23
	35	Cross-platform tool integration ability	22

Frequency analysis of indicator elements shows that common competency indicators such as subject value recognition and subject theoretical literacy appear more frequently (>29 times), which reflects the basic professional qualities possessed by moral education teachers; while discriminative competency indicators, such as technology platform application (23 times), learning behavior and thought analysis (23 times), and digital environment value sensitivity (22 times), although relatively low in frequency, jointly outline the outline of abilities necessary to adapt to smart learning environments. This set of capabilities covers the entire teaching process from tool operation, data analysis to value guidance, demonstrating the era’s requirements for the deep integration of instrumental rationality and value rationality in technology-enhanced teaching. Therefore, the final model construction must take into account both traditional subject literacy and new digital capabilities, forming a complete spectrum of capabilities that are both universal and situation-specific.

### Principal axis coding

Through continuous comparison and cluster analysis of the 35 initial conceptual categories obtained through open coding, 19 main categories were finally formed. These main categories are a further integration of discriminating competency indicators, namely digital environment value risk identification, digital teaching efficacy, digital lifelong learning, digital technology sensitivity, digital resource development, technology platform application, teaching data analysis, digital humanities narrative expression, digital teaching design, classroom organization and guidance, teaching evaluation reflection, digital ethics judgment, digital model creativity, human-computer collaborative teaching design, Critical thinking ability, online academic discourse influence, online educational community building ability, digital teacher ethics demonstration ability, and cross-cultural digital comparison ability. From this, the competency indicators and indicator elements of moral education courses in colleges and universities under the smart learning environment are determined.

### Selective coding

In the selective coding stage, through the analysis of categories at all levels and the identification of their internal logical relationships, based on the common terminology system in the field of international educational technology, a digital competency model for college moral education teachers in a smart learning environment was finally constructed, namely “value-oriented digital literacy—technology integration ability—Digital pedagogical competence—educational innovation thinking—digital citizen leadership”, as shown in Table 5. Each dimension provides conceptual definitions that go beyond operational descriptions to ensure that this model has a specific subject teaching context and can establish an effective academic dialogue with global digital competency research. This theoretical anchoring is the core methodological innovation of this study, which aims to go beyond simple empirical summary and contribute to the cross-cultural and interdisciplinary development of digital literacy theory. Among them, the ability to build an online education community is included in the dimension of digital citizen leadership. There are practical considerations behind this decision. Every comment and repost made by a teacher on the Internet may be amplified countless times and retained permanently. The demonstration effect brought by this digital imprint has already broken the boundaries of traditional classroom time and space. Teachers must realize that virtual interactions also require strict adherence to professional norms, and teacher ethics are extending from offline to every corner online. The critical ability of technological value is placed under the dimension of educational innovation thinking. The key is that this ability is not to teach teachers “how to use” technology, but to guide them to think about “why to use” and “what technology brings.” It is the most overlooked value examination link in educational innovation. When teachers begin to dialectically view the deep impact of technology on educating people, their innovations not only stop at the method level, but also touch upon the conscious construction of value rationality. This dimensional arrangement just highlights that truly in-depth innovation must include critical reflection.

**Table 5:** Digital competency indicators and definitions of teachers of ideological and political courses in colleges and universities under a smart learning environment.

Indicator	Concept definition	Element	Definition
A1 Value-oriented digital literacy.	Teachers systematically identify, analyze, evaluate and respond to value-sensitive content in the digital environment, and consciously embed a competency framework for ethical considerations in technology integration, emphasizing digital competency as a process of value judgment and ethical decision-making.	T1 Digital environment value risk discernment.	The ability to identify ideological risks in the digital environment.
		T2 Digital teaching efficacy.	The intrinsic motivation for pursuing digital teaching achievements.
		T3 Digital lifelong learning ability.	The ability to continuously learn independently, relying on digital resources.
		T4 Digital technology sensitivity.	The ability to perceive and explore the educational application of emerging technologies.
A2 Technology integration capabilities.	Teachers seamlessly incorporate digital tools, platforms, and resources into their subject-specific pedagogy, fostering systematic competencies for technology-enhanced instructional programs that harmoniously blend technology, teaching methodologies, and disciplinary content.	T5 Digital resource development capabilities.	The ability to develop and integrate digital teaching resources for ideological and political education.
		T6 Technology platform application power.	Proficient in operating various smart teaching platforms.
		T7 Teaching data analysis ability.	The ability to analyze and apply teaching data from the perspective of nurturing people.
		T8 Digital humanities narrative expression power.	The ability to tell ideological and political stories using digital technology.
A3 Digital pedagogical competence.	Teachers design, implement, evaluate and reflect on teaching activities based on digital environment, promote students’ ability system to construct meaning and develop higher-order thinking in situations, and emphasize the online balance between cognition, society and teaching.	T9 Digital teaching design capabilities.	The ability to design digital situational teaching plans.
		T10 Classroom organization and guidance	The ability to organize and guide the direction of digital classroom interaction.
		T11 Teaching assessment reflection ability.	The ability to evaluate and reflect on teaching using digital tools.
		T12 Digital ethics discrimination.	The ability to follow ethical norms in teaching data analysis.
A4 Educational innovation thinking.	Teachers use creative and critical thinking methods to explore new applications of digital technology, develop new teaching models, and emphasize solving real teaching problems through iterative design.	T13 Digital model creativity.	The ability to innovate digital ideological and political teaching models and methods.
		T14 Human-computer collaborative teaching design capabilities.	The ability to design personalized teaching plans through human-machine collaboration.
		T15 Critical thinking ability for problems.	The ability to examine the application of digital technology in a problem-oriented manner.
A5 Digital citizenship leadership.	Teachers practice, demonstrate and cultivate students’ abilities to use technology responsibly, participate in digital public life, and maintain digital ethics in the digital environment, and focus on the construction and demonstration of digital professional identity.	T16 The influence of online academic discourse.	The ability to lead the mainstream discourse direction in cyberspace.
		T17 Network education community construction power.	The organizational and coordination capabilities for building an online and offline collaborative education community.
		T18 Digital teacher ethics demonstration ability.	The ability to demonstrate professional ethics and morality in a network environment.
		T19 Cross-cultural number comparison power.	Compare the differences in digital ideological education models across countries.

To clearly present the theoretical framework of this model and facilitate international academic exchange, all constructs are anchored within cutting-edge international frameworks for scholarly dialogue. The five dimensions of the model encompass value orientation, technical operations, instructional implementation, innovative breakthroughs, and societal impact within educational contexts. Specifically, “Value-Oriented Digital Literacy” is grounded in UNESCO’s Global Framework for Digital Literacy and the EU DigComp Framework, emphasizing the development of digital literacy with value awareness and ethical reflection capabilities in educational settings; “Technological Integration Competence” draws on TPACK (Technological Pedagogical Content Knowledge) and SAMR (Substitution, Augmentation, Modification, Redefinition) theories, focusing on the organic integration of technology into moral education disciplines; “Digital Teaching Methodology Competence” builds upon the Community of Inquiry Model, Situative Learning Theory, and the EU DigCompEdu Framework, prioritizing the cultivation of subject-specific pedagogical expertise in online and blended environments; “Educational Innovation Mindset” references the EU Innovative Teaching Framework and ISTE Educator Standards, transforming pedagogical innovation into articulatable and developable behavioral competencies; “Digital Citizenship Leadership” integrates ISTE Standards and digital citizenship education theories, highlighting teachers’ professional demonstration, community co-construction, and public influence within digital environments. These interconnected dimensions collectively point toward a contextualized and integrated perspective on teacher digital competence development.

### Model validation

To validate the scientific rigor, reliability, and dimensional indicator weights of the competency model for moral education teachers in higher education within a smart learning environment, this study employed a questionnaire survey method to collect data through online platforms, conducting empirical testing and refinement of the theoretical model. To avoid conceptual overlap between dimensions, this research clearly defined the theoretical relationships among the five dimensions: A1 Value-oriented digital literacy serves as the foundational layer, providing value judgment benchmarks for other dimensions; technical integration ability (A2) and digital pedagogical competence (A3) constitute the practical implementation layer, where A2 represents input conditions and A3 embodies specific execution of teaching processes; educational innovative thinking (A4) and digital citizenship leadership (A5) form the reflective expansion layer, with A4 driving optimization and breakthroughs in teaching models while A5 extends capacity influence from classrooms to professional communities and public spaces. A1 and A4 emphasize internal cognition and thought patterns, A2 and A3 focus on external practical operations, and A5 bridges intrinsic beliefs with external societal impacts. This structural approach ensures each dimension maintains distinct theoretical positioning and practical orientation. Furthermore, high discriminant validity demonstrated in confirmatory factor analysis corroborates the independence of these dimensions.

The main content of the questionnaire was designed based on the teacher digital competency index elements and interpretations in Table 5, and was revised based on mature scales at home and abroad, combined with the characteristics of moral education teaching and smart learning application scenarios. After deletion, addition, and modification, 38 digital competency items were finally formed. A 5-point Likert scale was used to prepare the scale and score the questions, where 5 represents completely consistent, 4 represents relatively consistent, 3 represents somewhat consistent, 2 represents relatively inconsistent, and 1 represents completely inconsistent. With the help of the “Questionnaire Star” online platform, links are distributed to teachers from 15 universities in eastern, central and western China through the interpersonal relationships of teachers at the school. In addition, relevant information about college teachers who have research results in the field of digital ideological and political education was also collected through the Internet, and links were distributed to them to fill in their answers. Through statistics, it was found that a total of 388 questionnaires were filled out online and 375 were returned. After strict screening, after eliminating invalid questionnaires that were incompletely filled in, took less than 800 s to answer questions, and showed obvious regularity in the answers, A total of 292 valid questionnaires were finally collected, with an effective response rate of 75.25%, meeting the required sample size to item ratio for confirmatory factor analysis (CFA). The sample covered teachers of different genders, teaching seniority, disciplines, and professional titles. The gender distribution showed a nearly balanced male–female ratio, with males slightly overrepresented at 55.82%. Age distribution revealed that 79.45% of respondents were under 40 years old. Academic qualifications were predominantly doctoral degrees (72.26%). Lecturers constituted the majority of respondents (52.8%), while full professors accounted for only 10.26%. Teaching experience distribution showed 82.35% had 0–10 years of service, and 17.65% had more than 10 years. Among these, 194 cases were randomly assigned for exploratory factor analysis (EFA), and the remaining 98 cases were reserved for CFA, resulting in an approximate 2:1 sample allocation ratio between EFA and CFA samples.

**Figure 1:**
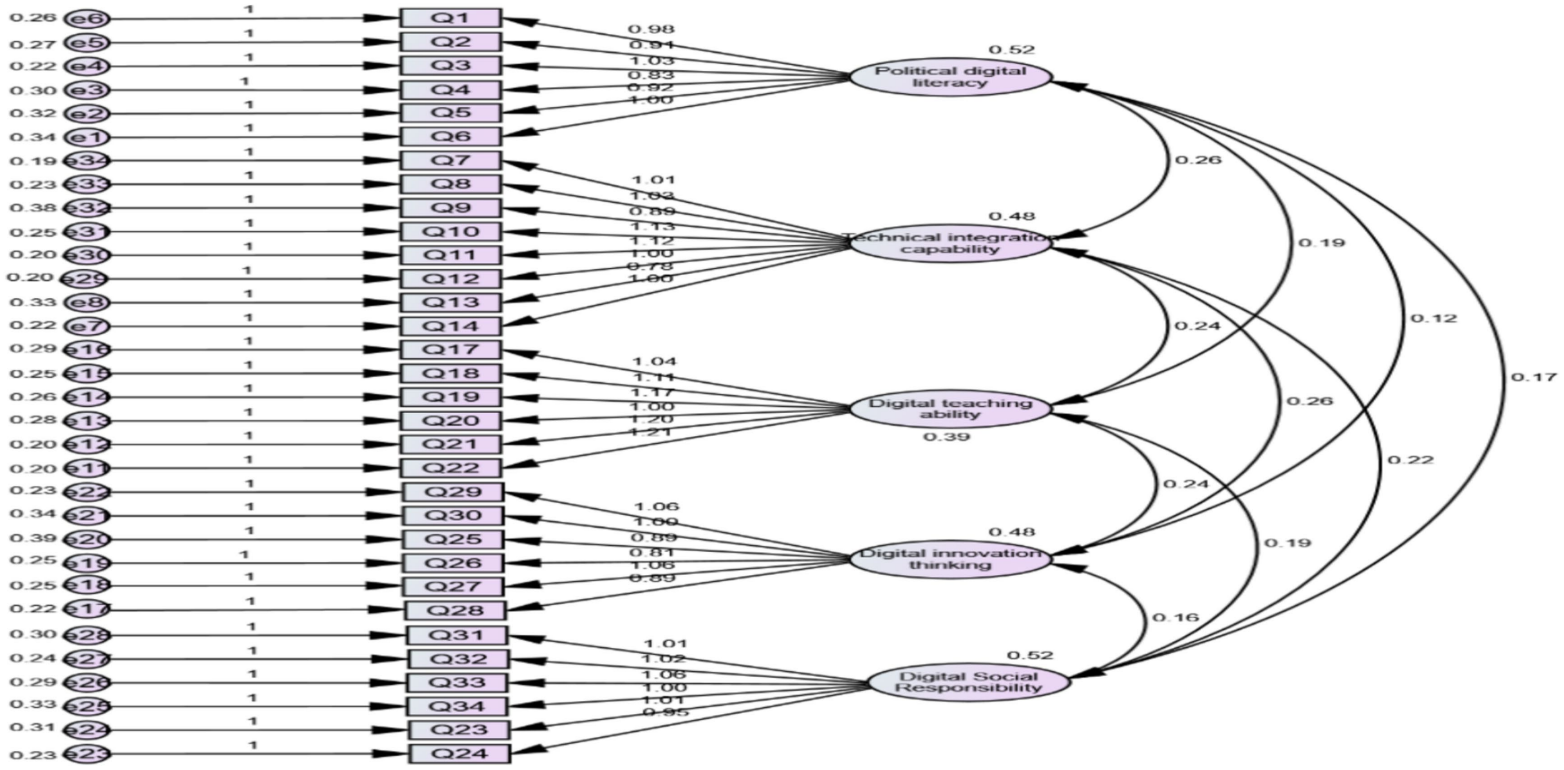
Structural equation modeling diagram.

To validate the validity of the initial scale, exploratory factor analysis (EFA) was conducted on the questionnaire data using SPSS 26.0, focusing on the Kaiser-Meyer-Olkin (KMO) measure and Bartlett’s test of sphericity. The EFA results indicated a KMO value of 0.927 and a statistically significant Bartlett’s test (*p* < 0.001), demonstrating the suitability of the data for exploratory factor analysis (Hair et al., 1995). Principal component analysis extracted five common factors with eigenvalues greater than 1, accounting for a cumulative variance contribution rate of 74.808%, which aligned with the five predefined categories in the theoretical model. This finding suggests that digital competence among moral education teachers exhibits a clear multidimensional structure rather than being limited to singular technical operational capabilities. The emergence of value-oriented digital literacy as an independent factor indicates that value judgment and technological application are complementary in teachers’ cognition, representing a critical challenge in digitalized moral education instruction. The highest factor loadings within the digital pedagogical capability dimension involved items related to instructional design and classroom organization, reflecting the application of pedagogical knowledge in technology integration within the TPACK framework. The identification of a digital innovative thinking factor demonstrates the developmental trajectory from technical application to pedagogical innovation, consistent with staged theories of teacher professional growth. However, the rotated component matrix revealed misclassifications requiring adjustments (see Figure 1). For instance, two items (Q15 and Q16) corresponding to T8 (digital humanities narrative expression ability) showed factor loadings below the 0.5 threshold (0.302 and 0.367 respectively) and were deleted. Similarly, cross-loadings exceeding 0.4 across factors A4 and A5 for two items under T19 (cross-cultural digital comparison ability) necessitated their removal. These discrepancies between the qualitative and quantitative phases are not anomalies but rather expected outcomes in mixed methods research. The qualitative phase, based on behavioral event interviews with 50 teachers, aimed to capture the full spectrum of potential competency indicators, intentionally adopting an inclusive approach to avoid omitting any relevant dimensions. This exploratory nature may have generated some indicators that, while contextually meaningful in specific interview narratives, did not demonstrate sufficient statistical consistency when measured across a larger, more diverse sample. The subsequent quantitative phase served to empirically validate and refine these qualitatively derived indicators, retaining only those that exhibited robust psychometric properties. Beyond item deletion, two indicators were reassigned to more appropriate dimensions based on both statistical evidence and theoretical alignment. T4 (digital technology sensitivity), originally assigned to A1 (value-oriented digital literacy), demonstrated a higher factor loading on A2 (technology integration capability). Given that T4 focuses on perceiving emerging technologies’ educational potential,a techno-operational sensitivity,it was relocated to A2, consistent with the technological knowledge component of the TPACK framework. Conversely, T12 (digital ethics discernment ability), originally under A3 (digital pedagogical competence), loaded more strongly on A5 (digital citizenship leadership). Theoretically, ethical considerations in teaching data analysis extend beyond classroom pedagogy to teachers’ public role as digital citizens and ethical leaders, justifying its relocation to A5. Subsequently, confirmatory factor analysis (CFA) examined whether items belonged to the five predefined primary categories. Analysis of the rotated component matrix using varimax rotation showed that most items exhibited factor loadings >0.6 on their respective primary factors, generally consistent with the initial category construction model. After these refinements, 17 initial categories corresponding to 34 items were retained in the final model.

After revising the initial model, 34 items were retained. Data collection was re-conducted according to the aforementioned requirements, and the samples were randomly divided into two groups. EFA analysis of the first group (194 questionnaires) yielded the following results: KMO value of 0.925 (*p* < 0.001), with 5 common factors extracted. After factor rotation using the maximum variance method, the percentages of variance explained by each factor were 15.295, 12.999, 12.912, 12.414, and 12.331% respectively, with a cumulative variance of 65.950%. This indicates that the five factors can adequately explain most information from the original variables. All corresponding items remained within their respective common factors after rotation, with factor loadings exceeding 0.65 and no significant cross-loading phenomena observed. These findings demonstrate that the revised scale exhibits satisfactory convergent validity. Reliability analysis revealed Cronbach’s *α* coefficients for the five dimensions—value-oriented digital literacy, technological integration competence, digital pedagogical capability, educational innovative thinking, and digital citizenship leadership as 0.890, 0.905, 0.912, 0.893, and 0.900 respectively, all exceeding 0.8. This confirms the excellent internal consistency reliability of the revised scale.

Subsequently, a second dataset comprising 98 questionnaires was employed to conduct confirmatory factor analysis on the revised competency model using AMOS 24.0. A note on the sample size is warranted here. While conventional guidelines often recommend at least 200 cases for structural equation modeling of this complexity, simulation research has shown that smaller samples can still be adequate when factor loadings are high and model fit is strong (Wolf et al., 2013). In the present study, the revised scale demonstrated high factor loadings, and the subsequent model fit indices proved to be excellent. Given these favorable statistical properties, the sample of 98 cases, though relatively modest, is considered acceptable for the confirmatory factor analysis reported here. The conceptual framework of all five dimensions was empirically supported by the data; all standardized regression coefficients exceeded 0.65 with high significance levels. The composite reliability (CR) values for the five dimensions ranged from 0.90 to 0.94 (CR > 0.9), while the average variance extracted (AVE) ranged between 0.60 and 0.68 (overall AVE > 0.6), demonstrating adequate convergent validity and internal consistency reliability of the scale. The exogenous variables corresponding to each of the five latent constructs exhibited strong correlation patterns, indicating the stability and reliability of the competency model. These results further validated that the relationships between latent variables and observed indicators in the model were essentially consistent with the theoretical framework. Additionally, all model fit indices reached satisfactory levels, the overall model fit was satisfactory (See Table 6). Specifically, the RMSEA value of 0.023, which is substantially below the critical threshold of 0.08, indicates that the theoretical construct adequately reflects the actual competency structure of teachers; the CFI value of 0.989 demonstrates a highly desirable improvement compared to the null model; and the χ^2^/df ratio of 1.051 approaching 1 suggests that the model achieves both parsimony and adequacy while avoiding overparameterization. The five competency dimensions exhibit complex interactions among technology, pedagogy, and values within effective teaching practices. Based on the theoretical model, each dimension is hypothesized to relate to different aspects of teaching practice. Value oriented digital literacy is theoretically associated with directional correctness in instruction; technology integration capability is conceptually linked to instructional efficiency; digital pedagogical competence is proposed to support student engagement; educational innovative thinking is theoretically associated with breakthroughs in teaching models; and digital citizenship leadership is proposed to relate to teachers’ professional demonstration and social influence. These theoretical pathways require empirical validation in future research, as shown in Figure 1.

**Table 6:** Results of the fitting degree test of the digital competence model for ideological and political course teachers in colleges and universities.

Statistical inspection quantity	Absolute fit index			Relative fit index		
	χ^2^/df	SRMR	RMSEA	IFI	TLI	CFI
The parameter fitting standard of this model	1.051	0.0592	0.023	0.989	0.988	0.989
	<3.0	<0.08	<0.08	>0.9	>0.9	>0.9

Other parameters: Chi-square value(χ^2^) = 476.947; Degree of freedom(df) = 454; *p* < 0.001.

In order to clarify the relative importance of each dimension in the model, this study can provide more targeted development suggestions for teachers by analyzing the index weights, and help educational managers and policy makers design more accurate teacher training and evaluation systems (Hair et al., 1995). Therefore, the indicator weights were estimated through principal component analysis method. First, the data were standardized and principal component analyzed. The results showed that the principal component characteristic roots of the five common factors were 5.102, 4.230, 4.179, 4.070 and 4.031, respectively. Then, the coefficient score is calculated through the component matrix loading coefficient, and then the principal component and the weight of each item are calculated respectively, that is, the weight of each dimension and its indicator is calculated. At the same time, calculate the score of each feature item in the entire model. The weight analysis results show that the weights of the five core categories of value-oriented digital literacy, technology integration capabilities, digital pedagogy capabilities, educational innovative thinking, and digital citizen leadership are 0.236, 0.196, 0.193, 0.188, and 0.187, respectively. The order of importance is ranked as follows: A1 Value-Oriented Digital Literacy > A2 Technological Integration Ability > A3 Digital Pedagogy Competence > A4 Educational Innovative Thinking > A5 Digital Civic Leadership. Among them, value-oriented digital literacy has the highest weight, confirming the principle that value takes precedence over technology in moral education teaching, and technology integration and application must serve educational goals; technology and pedagogy capabilities have similar weights, indicating that tool mastery and instructional design are equally important in smart teaching, and both affect teaching results; innovative thinking and civic leadership have a slightly lower weight, but this is in line with the gradual development of teachers’ abilities from classroom practice to community innovation, and from individual specialization to public influence. Finally, the model was improved again based on the results of factor analysis and weight analysis.

**Figure 2:**
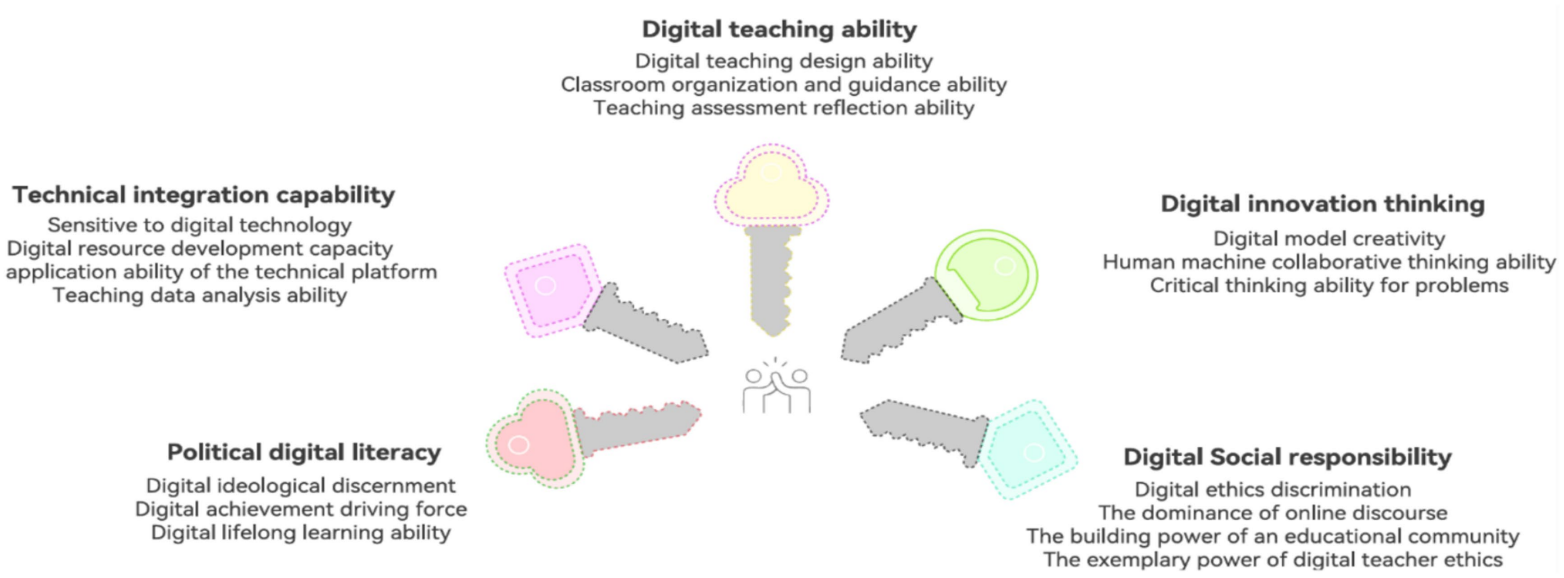
Digital competence model for ideological and political course teachers in colleges and universities.

Figure 2 presents a dynamic relational network formed by the interactions among five dimensions of the digital competence model for moral education teachers in higher education institutions. Positioned at the core, A1 Value-Oriented Digital Literacy vividly illustrates how teachers’ values guide technology selection, instructional design, innovation directions, and leadership behaviors in digitalized moral education teaching. Adjacent to it, A2 Technological Integration Capability provides the essential resource foundation for implementing A3 Digital Pedagogy, directly determining the depth and breadth of digital transformation in teaching practices. The two dimensions of A4 Educational Innovative Thinking and A5 Digital Citizenship Leadership radiate outward, signifying the expansion of teachers’ professional influence from closed classrooms to open public domains of professional learning. Particularly significant is the societal impact generated by A5 Digital Citizenship Leadership, which functions as critical environmental feedback that continuously shapes teachers’ value judgments and stimulates technological innovation. This creates an evolving, cyclically enhancing development loop. The nonlinear, cyclical interactions indicated by the arrows in the figure profoundly reflect the holistic nature, contextual sensitivity, and continuous evolution of digital competence within authentic educational settings.

## Discussion

The main theoretical contribution of this study is to propose a set of potential mechanisms through which the digital competency of college moral education teachers may influence teaching effectiveness, providing theoretical pathways for future empirical testing. The value filtering mechanism uses value-oriented digital literacy as a “filter” to screen the value appropriateness of technical tools and teaching content to ensure that technology integration does not deviate from the educational goal; the technology amplification mechanism uses technology integration capabilities to “amplify” high-quality teaching design and innovative ideas into scalable and replicable digital practices, breaking through the time and space limitations of traditional classrooms; the innovation diffusion mechanism uses educational innovation thinking to innovate teaching models and promote the diffusion of local teaching breakthroughs to systemic changes. The leadership demonstration mechanism uses digital citizen leadership to promote teachers’ online demonstration and community leadership, transforming individual abilities into collective practice, and forming a joint force in educating people. These mechanisms are proposed to help understand why pure technical training often has limited effects, and systematic capability development that integrates value, technology, teaching, innovation and leadership can produce substantial teaching improvements.

A1 Value-oriented digital literacy is the core of digital literacy for college moral education teachers. Its connotation has evolved from static knowledge and skills to a situational practical wisdom that makes value judgments in a complex intermediary environment composed of algorithms, interfaces and data flows. This is first reflected in the deepening of the value and risk identification of digital environments, that is, teachers need to deconstruct the cultural biases, value presets, and business logic that may exist behind seemingly neutral technical interfaces and massive information, and guide students to develop corresponding critical immunity. Secondly, the connotation of digital teaching efficacy has also been upgraded from mastering specific tools to an overall grasp of new ways of knowledge production and meaning negotiation in the digital age. It requires teachers to understand digital narratology, communication psychology and learning science to trigger interactive experiences of meaning construction. Thirdly, the essence of digital lifelong learning is the reconstruction of digital language capital, that is, understanding the generation rules of the Internet discourse field and transforming the theoretical system into expressions that conform to the laws of digital cognition. Generally speaking, this kind of literacy is highly value-laden. It runs through the entire process of teachers from technology selection to interactive design. Its training goal is to make a leap from “correctness of viewpoints” to more complex value wisdom. This enables teachers to skillfully identify discourse intentions, analyze logical fallacies, and analyze technological value loads in the complex chain of “teachers → technological intermediaries → students”, thereby releasing the critical and liberating functions of education in the digital space.

The discussion of A2 technology integration ability reveals that in the teaching of moral education subjects, technology integration follows the deep logic of dynamic balance between instrumental rationality and value rationality. The ability to develop digital resources is no longer a simple accumulation of materials, but has evolved into a knowledge graph construction ability based on semantic networks and digital humanities technology. Teachers achieve deep integration of content, technology and teaching significance by conducting semantic annotation and correlation analysis of teaching content. The essence of technology platform application power is to understand the platform as a technical ecology that builds cognitive situations and interactive ecology. This requires teachers to have insight into the architectural logic of different platforms, be able to transform abstract teaching designs into executable data processes, and make prudent technical choices based on educational goals in the process. The power of teaching data analysis constitutes a key closed loop for shifting from experience-driven to evidence-driven teaching. Teachers need to establish a data observation index system that conforms to the rules of subject learning, use learning analysis technology to identify students’ weak links in the process of conceptual understanding and value recognition, and ultimately transform data into precise educational interventions that promote value internalization.

A3 Digital pedagogy capabilities represent a paradigm reconfiguration from knowledge transfer to meaning negotiation. The key to teaching design is to use virtual simulation and other technologies to create a high-fidelity, low-risk experience and inquiry space based on situated learning and cognitive embodiment theory, so that abstract values can be made tangible, negotiable, and thinkable. The guidance of classroom organization requires teachers to be not only topic moderators, but also cultivators of digital interactive ecology. They need to use digital tools to cultivate an “inquiry-based community” that integrates cognitive existence, social existence and teaching existence, and guide constructive dialogue in the collision of multiple perspectives. The reflective ability of teaching evaluation and the discernment of digital ethics jointly ensure the quality and boundaries of the meaning negotiation process. The former ensures the accessibility of communication, and the latter protects the safety and ethics of communication. This teaching method ability ultimately points to the construction of a comprehensive assessment model that can integrate process data, emotional data and cognitive development data to reflect the special laws of value internalization, thereby supporting the continuous iteration of teaching.

A4 Educational innovation thinking is better understood as a generative mechanism, emerging from a dialectical interaction between “possibility thinking” and “constraint thinking,” rather than being solely reliant on the chance occurrence of individual inspiration. It is incumbent upon teachers to progressively reconstruct their teaching practices, anchored by clearly defined values. This necessitates that educators cultivate both “possibility thinking,” which envisions the potential of technology to expand cognitive boundaries, and “constraint thinking,” which takes into account practical ethical, institutional, and cultural constraints to ensure the robustness of innovations. The abilities of digital pattern creativity and human-machine collaborative instructional design exemplify “possibility thinking,” prompting teachers to experiment with new tools and methodologies. Inherently, critical problem-solving thinking embodies “constraint thinking” as it involves establishing and upholding fundamental norms of collaboration, respect, and rationality within open innovation processes. When teachers successfully balance these two modes of thinking, educational innovation evolves from a technology-driven serendipity to a sustainable professional practice cycle. This cycle is evidence-based and consistently aligned with educational objectives.

The proposal of A5 digital citizen leadership responds to the reshaping of teachers’ professional roles and responsibilities by the digital environment. Facing new challenges such as data privacy and algorithmic bias, teachers’ ethical practices need to extend from the classroom to the public space. In the classroom, they need to develop data ethics judgments oriented towards educating people; in professional communities, they need to co-build shared resources and formulate new norms for online collaboration; in the public sphere, they should undertake technical criticism and connect social responsibilities. To this end, we advocate a composite ethical framework that integrates virtue, obligation and consequentialist perspectives. At the same time, the Internet transforms teachers from “classroom authorities” into “key nodes in distributed networks.” Through cross-community knowledge sharing, ethical demonstration and ecological co-construction, teachers transform individual experience into collective educational wisdom. Furthermore, by participating in public practices such as digital content production and platform algorithm discussions, teachers can substantially influence the generation mechanism of digital culture, thereby significantly enhancing their visibility and influence in the digital public sphere while expanding their professional roles.

Although originating from the field of moral education within the Chinese higher education context, this framework offers potential references for humanities and social sciences instruction in similar settings. However, given the culturally situated nature of value oriented competencies, direct application to other disciplinary or cultural contexts, such as history, philosophy, or literature education in non-Chinese systems, would require careful contextual adaptation and further empirical validation. The key to successful adaptation lies in creative contextual interpretation of critical dimensions. For instance, A1 can evolve into “digital historical reasoning competence” within history pedagogy, while emphasizing “digital text interpretation literacy” in literary studies. Other dimensions such as technology integration and pedagogical innovation already demonstrate inherent versatility. When considering the application of this framework across different educational stages or cultural contexts, essential considerations include students’ cognitive development levels, local technological infrastructure, sociocultural variations in value perception, and institutional compatibility. The model’s modular architecture supports stable core principles with flexible peripheral implementation strategies. However, practitioners are advised to conduct situational analysis and small scale pilot validation before implementing the framework in new cultural or disciplinary settings, as the model’s empirical basis remains grounded in the Chinese moral education context. This dual capacity for abstraction from specific experiences and subsequent adaptation to diverse contexts embodies the theoretical vitality that educational models should possess. Furthermore, it provides disciplinary perspectives and heuristic analytical frameworks for addressing globally shared educational challenges like misinformation proliferation and value polarization.

## Conclusion, implications and limitations

This study, based on in-depth interviews with college moral education teachers regarding their practical narratives, employs grounded theory methodology for coding and theoretical construction. It proposes an analytical framework for digital competence among college moral education teachers. The framework reveals three interconnected competency levels: the value guidance level centers on A1, driving teachers to consistently adhere to disciplinary ethics and professional responsibilities as educators in technological applications; the practical operation level integrates A2 and A3, where the former manifests through utilizing intelligent tools to innovate teaching methods and construct immersive experiences, while the latter emphasizes analyzing and interpreting instructional data to achieve precise understanding of students’ learning processes and cognitive development; the reflective development level targets A4 and A5, requiring teachers not only to collaborate innovatively within multi-platform environments but also to maintain critical judgment regarding the educational value of technology, thereby playing proactive leadership roles in professional communities and public spheres. The advancement of smart education imposes practical demands on upholding fundamental principles while innovating moral education disciplines, with enhancing teacher digital competence serving as the key response to these requirements.

The essence of smart education goes far beyond the application of technology. It prompts us to re-examine the inherent relationship of the teaching process. For moral education teachers, this means that their role is no longer a knowledge imparter, but a builder of learning ecology and a guide for meaning negotiation. The key is to integrate intelligent technology into the inner context of subject teaching, especially the process of cultivating critical thinking, conducting ethical discussions, and generating value meanings. If we only stay at the technical level, we will easily fall into the cycle of instrumentalism and deviate from the goal of promoting deep learning. In teaching, teachers need to transform critical thinking, digital citizenship literacy and other training goals into concrete and perceptible classroom practices. This provides the possibility for learning tasks that stimulate thinking and interactive dialogues that contain value care. Only in this way can digital teaching radiate the interactive vitality and humanistic warmth it deserves while maintaining the depth of thought. The digital age has put forward higher requirements for teachers’ digital practical ability. This is not the development of a single skill, but a literacy ecology that needs to be systematically cultivated. Colleges and universities should pay attention to the integration of intelligent technology and teaching situations. First, teachers not only need to master VR、AR, learn how to operate analysis tools, and more importantly, it is necessary to use these tools to create immersive experience scenes, or build an exploration environment that supports students to deconstruct complex problems layer by layer, so that technology can truly serve the cognitive process. Second, it emphasizes the shift in data-based educational insights and evaluation. Teachers must learn to identify students’ cognitive characteristics, thinking habits and learning disabilities from multi-dimensional data such as classroom interaction records, online discussion traces, and homework feedback, so as to promote teaching towards a personalized guidance teaching paradigm. Third, promote an open and collaborative teaching and research innovation culture. Teachers are encouraged to break through organizational boundaries, actively participate in cross-school and cross-regional digital teaching and research networks, and continue to iterate their own educational practices based on collective wisdom through collaborative development of resources, collaborative design of teaching cases, and joint action research. In an educational environment where technology is accelerating iteration, teachers should consciously become reflectors of ethical practice and guides of digital literacy, placing value considerations at the core of technology integration. When teachers handle student data, they must strictly follow privacy protection guidelines and implement the principle of informed consent throughout; when faced with intelligent technologies such as algorithm recommendations, they must make good use of their personalized support functions, it is also necessary to guide students in jointly examining potential ethical dilemmas such as information cocoons and algorithmic bias. Moreover, we should proactively use digital media to create educational content that contains ideological depth, solid evidence, and humanistic care, and cultivate a public culture of rational dialogue in the complex online context. These practices jointly point to the fundamental purpose of education, that is, all technological applications should serve the comprehensive development of people, rather than letting people be coerced by technological logic. Only in this way can smart education truly carry the essential proposition of “what kind of people to cultivate”.

This study still has some limitations, which provide possible directions for subsequent research. First, the research sampling is mainly based on the Chinese university environment, specifically within the context of moral education in Fujian Province. Although the model itself emphasizes theoretical transferability, its adaptability in different countries, cultural backgrounds, other disciplinary settings (e.g., history, philosophy, literature), and even other regions within China requires further empirical verification. Future cross-cultural and cross-disciplinary validation studies are needed to examine the model’s measurement invariance and contextual relevance. Secondly, data collection mainly relies on self-reported questionnaires, which may be subject to social desirability bias. In the context of digital competence and moral education, respondents might rate themselves more favorably than their actual classroom practices reflect. To mitigate this bias, the questionnaire was administered anonymously, and participants were assured that their responses would be used solely for research purposes with no impact on their professional evaluation. Additionally, reverse-coded items were included in the initial scale to detect careless or biased response patterns. The qualitative interview data also served as a triangulation source, providing cross-validation for the self-reported measures. Nevertheless, future research should integrate multi-source data such as classroom field observations, teaching file analysis, or student evaluations to capture teachers’ actual teaching behaviors more objectively, thereby further reducing social desirability bias and improving the ecological validity of the findings. Third, the currently constructed digital competency model of moral education teachers presents a relatively static structure. Future research can further use in-depth case studies to reveal how these competency dimensions interact and develop dynamically during the professional growth of teachers. Finally, while this study validates the structural validity of the competency model, it does not directly measure the model’s impact on student learning outcomes, including the development of critical thinking skills or the acquisition of digital citizenship. Future research should empirically test the hypothesized links between this competency framework and indicators of teaching effectiveness.

## Data Availability

The original contributions presented in the study are included in the article/supplementary material, further inquiries can be directed to the corresponding author.
